# Fostering Academic Engagement in Post-graduate Students: Assessing the Role of Positive Emotions, Positive Psychology, and Stress

**DOI:** 10.3389/fpsyg.2022.920395

**Published:** 2022-08-09

**Authors:** Muhammad Shoaib Saleem, Ahmad Shahrul Nizam Isha, Maheen Iqbal Awan, Yuzana Binti Yusop, Gehad Mohammed Ahmed Naji

**Affiliations:** ^1^Department of Management and Humanities, University of Technology Petronas, Tronoh, Malaysia; ^2^Faculty of Medicine, Sultan Zainal Abidin University, Kuala Terengganu, Malaysia

**Keywords:** positive emotions, psychological capital, study engagement, stress, post-graduate students

## Abstract

**Aim:**

The current study attempted to assess the effect of positive emotion on post-graduate students’ psychological capital (PsyCap) as well as on their academic engagement behavior. Also, the direct relationship between PsyCap and academic engagement behavior was assessed alongside the presence of Stress as a moderating variable between PsyCap and academic engagement behavior amongst post-graduate students in Malaysia.

**Materials and Methods:**

A self-administered questionnaire was used for data collection from 373 post-graduate Ph.D. students registered in various universities throughout Malaysia with a non-probabilistic sampling technique. Research respondents belonged to management, humanities, engineering, computer science, and health sciences domains, and they responded through a questionnaire copy. Statistically, structural equation modeling (SEM) was applied to evaluate confirmatory factor analysis (CFA), reliability analysis, validity analysis, measurement model, structural model, and path analysis. Furthermore, the (5000) bootstrapping approach was utilized to test the final model.

**Results:**

For the hypothesized model, our results confirmed that positive emotions had a positive and significant effect on students’ psychological capita as well as on their academic engagement behavior. Further, PsyCap also had a positive and significant effect on academic engagement behavior. Our results also reported that stress as a moderating variable has a negative and deteriorating effect on the relationship between PsyCap and the academic engagement of students.

**Conclusion:**

The study’s findings support the theoretical assumption that positive emotions help individuals generate cognitive resources, which in turn help them manage their engagement behavioral requirements. However, the stress caused by their study needs may deplete their psychological resources, consequently influencing their academic engagement behavior. Interventions like personal coaching/counseling, appropriate follow-up, and flexible goal settings with other measures may help post-graduate students in achieving their daunting tasks.

## Introduction

With the advent of positive psychology, its application has seen tremendous growth in different industrial sectors, be it production, services, or even the educational sector (Avey et al., 2010; He et al., 2019; Carmona-Halty et al., 2021; Kang et al., 2021; Saleem, 2022). For the educational sector, contemporary research has witnessed the augmentation of students’ academic engagement through their positive emotions and positive psychology (Carmona-Halty et al., 2021; Dewaele and Li, 2021; Luthans et al., 2021). The notion of positive psychology gave birth to the construct known as psychological capital (PsyCap), an important factor that can improve efficiency and competitiveness (Luthans et al., 2004) as well as the academic outcomes (Luthans et al., 2012). However, prior research has covered mostly high-school/sophomore students (Carmona-Halty et al., 2021) and university undergraduate students (Luthans et al., 2014, 2021; Kang et al., 2021; Saman and Wirawan, 2021), with no studies conducted on post-graduate research students, i.e., Ph.D. scholars.

Talking specifically about high-school and undergraduate university students, who are provided with the classroom and physical teaching environment, prior research suggests that inculcating happy emotions in their classrooms may yield resilience, mindfulness, and physical health for them (Fredrickson et al., 2008). Whereas, when we talk about the post-graduate student’s educational journey, it is much different than undergraduate and high-school students in terms of the aspects such as; length of the degree, course work, examination, assessment, educational outcomes, teaching methodology, and so on (Eckersley et al., 2016; El-Ghoroury et al., 2012). Such difference between the undergraduate and post-graduate students’ academic tenure brings new challenges for them. Out of many issues, one of the most possible prominent issues that post-graduate students may face is a sense of social and intellectual isolation and self-dependency mostly in every aspect of their studies (Hortsmanshof and Conrad, 2003; Ali and Kohun, 2006; Essa, 2011; Brown et al., 2013; Al-Kumaim et al., 2021; Ramchander, 2022), and that may also trigger dissatisfaction, delay and a probable withdrawal from their academic objectives (Essa, 2011, p. 20; Brown, 2013; Haque et al., 2017). Since post-graduate students are perceived as lone venturers, hence the notion of isolation and consequent stress is much more relevant for them (Wisker et al., 2007). Further, post-graduate students, be it male or female, frequently or most of the time work alone, with few or no other persons on their project (Conrad and Phillips, 1995; Hortsmanshof and Conrad, 2003). Also, this occurs when friends or family members work in offices and teams, enjoying a significantly more social aspect of their life and this, possibly, may lead them to loneliness, a lack of drive, and the worry that no one understands or can relate to their difficulties (Brown, 2013; Haque et al., 2017). Working in isolation and mostly on their own is mainly the key difference between undergraduate and post-graduate students, alongside the amount of scholarly and scientific research outcomes that are expected from post-graduate students, which adds a burden to their educational journey (Robins, 2008; Ramli et al., 2021). Having said this, it is much expected that post-graduate students may feel less enthusiastic and find themselves troubled while completing their daunting studies (Essa, 2011; Al-Kumaim et al., 2021; Ramchander, 2022), hence the need to have positive emotions as well as enough psychological resources to accomplish this task seems much relevant to them as well to their academic engagement behavior (Pascoe et al., 2020). Literature also upholds that positive emotions help sustain prosocial behavior which may regularize emotions to the prime levels (Mesurado et al., 2021). Nonetheless, our study will help to advocate the application of positive emotions, academic PsyCap, and academic engagement in a different higher educational context. Moreover, there has been a surge of research inquiring into the role of emotions in academic settings in recent years, e.g., especially how emotions impact students’ engagement and learning (Linnenbrink-Garcia and Pekrun, 2011), whereas students with more positive emotions are expected to hold more positive cognitive resources like PsyCap (Carmona-Halty et al., 2021), and this may, in turn, lead them toward academic engagement behavior. In doing so, we will study the education-related positive emotions as a predictor of academic PsyCap leading toward possible academic engagement for post-graduate students of multiple disciplines.

While stress is an integral part of students’ life, the research posited that its presence in the academic journey for students is gaining more and more prominence (Beiter et al., 2015). With impending deadlines, large-scale projects, and considerable personal investment by post-graduate students, they may be quite stressful. This is accentuated by the notion that everything depends on you and only you, enhancing the highs and decreasing the lows. Post-graduate students have been found to have substantial levels of mental issues, which are most likely related to the high levels of stress they face (Bazrafkan et al., 2016; Wang et al., 2019). Also, pressure to obtain the highest qualification may yield stress for post-graduate students, as it is expected to put them under different kinds of mental strains, e.g., pressure of deadlines, pressure to produce quality publications, financial constraints, and uncertainty about the future, etc. (Beiter et al., 2015; Vasugi and Hassan, 2019). Having said this, it is to expect that the presence of stress for the post-graduate student will deplete their positive psychological resources, i.e. (PsyCap), consequently reducing their effectiveness in their academic engagement behavior.

Furthermore, work engagement is defined as one’s affective, cognitive, and behavioral components that are tied to the workplace or one’s professions (Schaufeli and Bakker, 2004; Schaufeli et al., 2006). The notion of work engagement has been extended to educational settings such as high schools and colleges to examine its potential outcomes in various situations (Fredrickson, 2004; Carmona-Halty et al., 2021). Evidence revealed that academic engagement includes traits such as having positive and gratifying study-related engagement based on one’s vigor, dedication, and absorptions. In contrast, the utilization of engagement construct as an academic engagement, particularly for post-graduate students, is essentially non-existent in the literature. Because post-graduate students typically study in isolation and rely significantly on their talents and capacities, thereby they may have issues such as stress, decreased motivation, boredom, and helplessness, hence measures that can enhance or predict their academic engagement are essential to study.

Our research connects both ends of the suggested research model using three separate theories for predictor as well as the outcome variables. To expound on the link between academic emotions and PsyCap, we will opt to broaden and build a theory, known as (B&B) (Fredrickson, 2004), which will explain how positive emotions might impact an individual’s cognitive resources, ultimately resulting in academic engagement. Secondly, to assess the association between PsyCap and academic engagement, we will be opting for the job demand-resources theory (Bakker and Demerouti, 2014), which proposes the positive association between resources and engagement behavior. In addition to aforesaid, the stress for post-graduate students also plays a key role throughout their study tenure, therefore we will be assessing its impact between PsyCap and academic engagement behavior under the purview of Conservation of resource theory (COR) (Hobfoll, 1989).

In summary, this research is intended to see positive emotions as a predictor of individuals’ PsyCap as well as the direct effect of PsyCap on academic engagement will also be assessed. From an analysis point of view, Structural equation modeling (SEM) will be used to statistically evaluate confirmatory factor analysis (CFA), reliability analysis, validity analysis, measurement model, structural model, and path analysis. Also, our research is significant to contemporary literature, as it may provide some interesting insights from the post-graduate students’ perspective as to how emotions play their part in higher education, as well as how their level of stress threatens their cognitive resources, which may, in turn, deter their academic engagement.

## Theoretical Background of the Constructs

### Academics Related Positive Emotions and Academic Psychological Capital

Someone or something is always responsible for eliciting positive emotions. Positive feelings are based on Lazarus (1991) description of emotions as a “reflection of a person’s judgment of their environment.” Positive emotion research has grown in popularity since the advent of Positive Psychology, and the release of the B&B theory marked a watershed moment in its evolution (Fredrickson, 1998). The B “broaden hypothesis” and the B “build hypothesis” are the two primary hypotheses specified in this theory. Positive emotions, according to the broad hypothesis, momentarily “broaden” people’s attention and thoughts, allowing them to access a broader range of ideas. According to the second hypothesis, these broadened perspectives enable people to find and “develop” crucial personal resources (Fredrickson, 2001). In our study, students’ positive emotions are manifested in a variety of ways, including positive feelings, happiness, joy, and satisfaction. Positive emotions, according to Slåtten et al. (2021), broaden and enlarge pupils’ attention and cognition for learning. Students’ attention and cognition for learning would be limited if they had a lower level of positive or negative emotions.

Psychological Capital is a cognitive component of the mind. PsyCap, which derives from positive psychology, focuses on an individual’s intrinsic qualities and positive resources that can be capitalized on or exploited. However, there is reason to believe that positive emotions will be favorably associated with PsyCap. In this study, PsyCap refers to an individual’s positive psychological state of development, which is defined as: “having confidence (self-efficacy) to take on and put in the necessary effort to succeed at challenging tasks; making a positive attribution (optimism) about succeeding now and in the future; persevering toward goals and, when necessary, redirecting paths to goals (hope) to succeed; and when beset by problems and adversity sustaining and bouncing back and even beyond (resilience) to attain success” (Luthans et al., 2007a,b; Luthans and Youssef, 2007). The affect component of the mind (positive emotions) can promote growth in the PsyCap component of the mind, which refers to the cognitive component of the mind. Positive emotion mechanisms, according to Slåtten et al. (2021), operate as enablers to directly enhance the PsyCap.

The experience of positive emotions is vital for comprehending the emergence of personal resources, according to empirical research. It was discovered that positive emotions related to the study are directly tied to PsyCap. This suggests that pupils who have a higher frequency of positive emotions in their studies are more likely to report high PsyCap levels (Carmona–Halty et al., 2019). More specifically, study-related positive emotions may contribute to the development of particular behaviors contained in the mental capital construct, such as flexibility and efficiency in learning, as well as resilience or perseverance in the face of stress and failure (Carmona–Halty et al., 2019). Another study discovered that students who experienced positive emotions related to their academics more frequently were more likely to report higher levels of PsyCap (Carmona-Halty et al., 2021). Because the academic PsyCap construct is important in the educational setting, and positive emotions are a significant variable related to academic PsyCap, hence we hypothesize that:

**H_1_:** Academics-related positive emotions positively influence academic PsyCap.

### Academic Psychological Capital and Academic Engagement

Because engaged learning is a goal that most educators strive for in their classrooms or schools, it has received extensive research in the educational industry. Among the different definitions of engagement, engagement in this study refers to the amount of cognitive effort, behavioral participation, and emotional quality connected with a student’s active involvement in learning (Fredrickson, 2004). PsyCap has a positive relationship with study engagement, especially when university students face high challenge demands (Siu et al., 2004). The authors went on to say that in a university setting, persons with a high personal resource of PsyCap should be intrinsically driven to study because they are drawn to the study subject itself, and hence feel more concentrated, dedicated, and full of vitality, and thus more engaged in study. Another study discovered that PsyCap increases an individual’s motivation to learn and is a critical resource for learning empowerment, which ultimately improves learning engagement (You, 2016).

According to Datu and Valdez (2016), students who believe in a combination of hope, optimism, resilience, and self-efficacy (PsyCap) may actively participate in numerous classroom tasks (behavioral engagement) and like undertaking academic work (emotional engagement). Similarly, Luthans et al. (2021) agreed that successful students have higher degrees of hope, efficacy, resilience, and optimism when pursuing positive academic performance outcomes. Furthermore, it was discovered that students’ PsyCap is vital for students’ academic engagement and the accomplishment of emotions. From a theoretical perspective, job-demand resource theory proposes that person-oriented resources can enhance engagement, but can also help to overcome job demand. Whereas, PsyCap is a personal resource that may help individuals to accumulate more personal resources to display engagement behavior. Therefore, we posit that:

**H_2_:** Academic PsyCap positively influences Academic engagement.

### Academics Related Positive Emotions and Study Engagement

Students’ study engagement may be increased by focusing on improving their positive emotions and personal resources. Positive emotions, according to Ouweneel et al. (2013), can eventually lead to involvement through strengthening personal resources such as instilling a sense of positivity toward students themselves, their academics, and the future. Another study found that happy emotions had a close relationship with study engagement. Because positive emotions in the classroom appear to increase students’ trust in their abilities to achieve their goals and increase students’ commitment to the task requested, researchers elaborated that positive academic emotions allow students to enjoy the academic task they are executing and activate their academic engagement (Oriol-Granado et al., 2017).

Furthermore, the findings of the Denovan et al. (2020) investigation supported the B&B Theory. They indicated that students will boost their resources as a result of feeling positive emotions, which will lead to increased wellbeing and academic engagement over time. Another study found that happy emotions were associated with increased levels of study engagement (Rodríguez-Muñoz et al., 2021). Based on the aforesaid, we hypothesize:

**H_3_:** Academics-related positive emotions positively influence study engagement.

### Stress as a Moderator

Academics are an important part of any college student’s life, and students who do not have a positive attitude toward academic goals may suffer from devastating bouts of stress. Completing grade requirements, taking tests, learning a large amount of material, and managing time have all been identified as major sources of stress for students. A variety of academic stressors can cause stress in a student’s academic environment (Misra and Castillo, 2004). Literature also upholds that the ultimate goal of education is to become educated, find work after graduation, and achieve some level of personal achievement. As a result, it is unsurprising, if not logical, that academics, success, post-graduation plans, and finances were the top sources of concern (Beiter et al., 2015). Talking about stress for students, Camacho et al. (2016) assessed stress in college students using the Depression, Anxiety, and Stress Scale commonly known as (DASS-21). While talking about stress, school and university management must develop programs that teach students how to use active, efficient, and functional coping techniques (such as problem-solving, cognitive restructuring, expressing emotions, and social support) to assist them to deal with stressful conditions associated with higher education. Students who can handle difficult situations feel stronger, more involved, and more immersed in their studies, which may improve their academic performance (Vizoso et al., 2018). Another study discovered that stress had an impact on pupils’ academic involvement (Van Ryzin and Roseth, 2021). Specifically talking about stress for post-graduate students, stress such as time management, funding issues, conflicts with the research supervisor, stress and anxiety, work-life balance, in some cases lack of institutional or personal support, and the uncertainty about one’s future is expected to cause depletion of one’s psychological resources, which may help him/her to stay focused and productive. Having said this, in our case we expect to see that stress negatively affects the relationship between PsyCap (predictor) and academic engagement (an outcome variable). Based on this, we propose:

**H_4_:** Stress negatively affects the relationship between academic PsyCap and study engagement.

Based on the literature and discussion above we have formulized the following research framework to be tested in post-graduate students in Malaysia:

## Methodology

### Instrument Structure and Design

Overall the questionnaire was comprised of five different sections such as demographics, positive emotions, PsyCap, stress, and study engagement. Every statement of the questionnaire was discussed with field research experts to get additional insights into the validity of the instrument. Face validity of the research instrument was attained through this process. Minor changes were made to the questionnaire wherever necessary, as well as text and wording were thoroughly reviewed keeping in view the conversation norms of post-graduate students. Further, the research team also met around six post-graduate students to discuss the questionnaire to assess the content validity and apprehension of the measurement and respondents did not report any difficulty in the comprehension of the questionnaire survey. The average time to fill the questionnaire was also calculated through six respondents, i.e., approximately 15 min.

### Measures Used

For this study, an already developed scale by Diener et al. (2010) commonly known as the “Scale of Positive and Negative Emotions” (SPANE) was adapted according to the Malaysian context. This scale contains overall twelve items, whereas six represent positive emotions and six represent negative emotional experiences. We used a 5-point Likert scale from 1 (rarely) to 5 (often) for measurement. Since we are looking at the predictive power of positive emotions toward academic PsyCap, thus we used only six indicators of positive emotional experiences according to the education context, with one of the example statements, “I feel positive about my studies.” Prior studies have reported a reliability value higher than 0.85 (Diener et al., 2010; Carmona-Halty et al., 2021), whereas in our case, Cronbach’s alpha was 0.933, which shows strong reliability.

To measure the academic PsyCap of post-graduate students, we used an already developed measure of PsyCap questionnaire-12 known as “PCQ-12” (Luthans et al., 2007b; Avey et al., 2011). This scale is the shorter version of PsyCap measurement for the academic sector containing three items for efficacy, four items for hope, three items for resilience, and two items for optimism. Responses were recorded on a 5-point Likert scale ranging from 1 (strongly disagree) to 5 (strongly agree). Some of the sample statements are; “I feel confident in representing my educational areas in meetings with my peers/groups/same level students,” “I feel confident helping to set goals in my study area,” and “I usually take stressful things in stride,” and “I always look on the bright side of things regarding my studies.” Cronbach’s alpha of the scale was 0.951, showing consistency among responses.

We used a shorter version of the work engagement scale by Schaufeli et al. (2006) previously known as Utrecht Work Engagement Scale (UWES-9) for our academic research context commonly known as. Overall the questionnaire contained nine statements, and in our case, since we measured two core dimensions of the academic engagement, i.e., vigor and dedication, therefore we used only six statements representing three for each dimension. We used a 5-point Likert scale to study engagement behavior ranging from 1-(Strongly disagree) to 5-(strongly agree). Some example statements were; “in my studies, I feel strong and vigorous,” and “I am enthusiastic about my studies.” Cronbach’s alpha of the scale was 0.927, showing consistency among responses.

To measure the stress amongst students, we used a scale developed by prior researchers and commonly known as Depression Anxiety and Stress Scale (DASS-21) (Lovibond and Lovibond, 1996), which was further validated by researchers (Osman et al., 2012) in the educational context. The scale represents three distinct dimensions of depression, anxiety, and stress. Since we are only interested to observe the effect of stress between PsyCap and study engagement, we took only one dimension from the scale, i.e., stress. We used a 5-point Likert scale ranging from 1-(Which did not apply to me at all) to 5-(Applied most of the time). A few example statement for stress includes, “I found it difficult to relax,” “I found myself getting agitated,” and “I tended to overreact to situations.” Cronbach’s alpha of the scale was 0.90, showing consistency of the received responses.

### Ethical Considerations

Our study was cross-sectional. All respondents were assured about the anonymity and privacy of their responses. In doing so consent form was signed by respondents in advance. Since the participation was voluntary in this research. We did not perform any sort of medical or educational test on our respondents (cognitive, diagnostic, or aptitude), as well as no specific procedure or observation of public (respondents’) behavior, was carried out, thus our study does not require ethical committee approval. Further, we did not use or ask for any private or identifiable information from respondents at any level. We also do not foresee any risk of harm or injury to our respondents through participation in our research.

### Data Collection and Data Analysis

Our respondents were post-graduate students enrolled in full-time Ph.D. programmes at Malaysian private universities in a variety of disciplines. Because we did not have an exhaustive list of our study population, we used non-probabilistic convenience sampling and snowball sampling techniques to collect data (Cooper and Schindler, 2014). It took about 6 months to collect the data because the responses were physically collected from various states in West Malaysia. Participants in our survey came from the fields of management, humanities, engineering, computer science, and health sciences. The respondents were given a hard copy of the questionnaire, and responses were collected based on their convenience and availability. All necessary guidance and support were provided while completing the questionnaire. The research team collected all the responses on the spot.

We gathered responses from active post-graduate university students admitted to 23 different private universities across multiple states. A total of 400 questionnaires were distributed, with 393 completed, resulting in a response rate of more than 90%. We had 373 final responses for final analysis after performing an initial evaluation of received responses and eliminating inappropriate, incomplete, and inconsistent responses. In terms of gender, 56% of our respondents were male students and 44% were females. Respondents came from a variety of faculties, including 35% from management sciences, 15% from humanities, 30% from engineering, 12% from computer sciences, and 8% from health sciences. Our respondents’ average age ranged from 29 to 36 years.

To run the statistical analysis, i.e., descriptive and reliability, we used SPSS Version 21. Further to test the convergent, discriminant validity, measurement model, and structural model fit we used SPSS Amos Version 21. To test the hypothesized model and moderation analysis we used SMART PLS. Our model is based on the SEM technique, which is a nominal research analysis approach (Saleem et al., 2021a,b). We evaluate the goodness of fit (GFI) for both measurement and structural models *via* indexes such as: “RMSEA = root-mean-square error of approximation,” “GFI = goodness-of-fit index,” “AGFI = adjusted goodness-of-fit index,” “NFI = Bentler-Bonett Normed Fit Index,” “TLI = The Tacker-Lewis index,” “CFI = Comparative fit index,” “Parsimony normed-fit index,” “NC = normed X^2^ (i.e., X^2^/degree of freedom).” To assess the overall convergent validity for constructs and their related items, we used parameters such as; standardized factor loadings (SFL >0.60), Average variance extracted (AVE >0.50), and Composite reliability (CR >70). Further, the discriminant validity of all variables was also computed through a comparison between the correlation coefficient of all variables and its subsequent comparison with the AVE (Bagozzi and Yi, 1988).

## Results

### Convergent and Discriminant Validity Analysis

Based on our findings, all variables resulted according to the defined criterion for acceptance, e.g., positive emotions, SFL ranged from 0.85 to 0.88, with CR = 0.947 and AVE = 0.699, PsyCap; SFL ranged from 0.78 to 0.82, with CR = 0.957 and AVE = 0.618, Stress; SFL ranged from 0.75 to 0.80, with CR = 0.92 and AVE = 0.562, and lastly for Study engagement; SFL ranged from 0.84 to 0.86, with CR = 0.943 and AVE = 0.679, (Bagozzi and Yi, 1988). The discriminant validity for all study variables was achieved, as presented in the Table 1.

**TABLE 1:** Discriminant validity.

Constructs	AVE	MSV	MaxR (H)	Positive emotions	PsyCap	Study engage	Stress	Skewness	Kurtosis
Positive emotions	0.699	0.319	0.933	**0.836**				−0.94	−0.08
PsyCap	0.618	0.319	0.951	0.565***	**0.786**			−0.78	−0.18
Study engagement	0.679	0.282	0.927	0.531***	0.421***	**0.824**		−0.58	−0.73
Stress	0.562	0.02	0.90	0.018	−0.018	0.140**	**0.749**	−1.01	0.65

**p < 0.010, ***p < 0.001. MSV, maximum shared variance; MaxR (H), mcDonald construct reliability. Bold values are the square root of the average variance extracted (AVE) with the correlation of latent constructs.

### Measurement and Structural Model

We assessed both measurement and structural models for all of the study variables, i.e., positive emotions, PsyCap, study engagement, and stress through different fitness indexes such as absolute, incremental, and parsimonious fit indices. Measures like RMSEA, GFI, AGFI, TLI, CFI, NFI, and CMIN/DF (χ^2^/degree of freedom) were utilized (Hu and Bentler, 1999). Our retrieved data were consistent with the established threshold. The measurement model and structural model outcomes are shown in Table 2, in which it is clear how our results have indicated an adequate good fit against the CFA.

**TABLE 2:** Measurement model and structural model validity.

	Variables	RMSEA	GFI	NFI	AGFI	CFI	TLI	CMIN
Measurement model results	Positive emotions	0.02	0.994	0.996	0.986	0.99	0.99	1.225
	PsyCap	0.001	0.987	0.991	0.981	0.99	0.97	0.86
	Study engagement	0.044	0.989	0.992	0.975	0.996	0.996	2.104
	Stress	0.01	0.994	0.994	0.988	0.97	0.96	0.851
Structural model results	Four study variables loaded onto four separate factors	0.006	0.954	0.966	0.947	0.998	0.999	1.021

*RMSEA, root mean square error of estimation; GFI, goodness of fit; NFI, normed fit index; AGFI, adjusted goodness of fit; CFI, comparative fit index; TLI, tucker lewis index; CMIN, chi-square.*

### Hypothesis Testing

**FIGURE 1:**
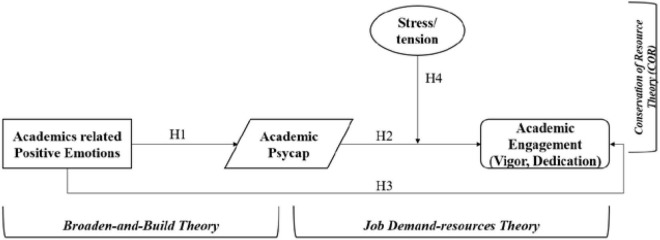
Proposed research framework.

To test the hypothesized model in Figure 1, we used the SEM approach. Our two outcome variables; PsyCap and study engagement were well predicted or explained by their predictors, i.e., PsyCap (28% variance explained through predictor) with the self-error variance of 72%, and study engagement (33% variance explained through predictor) with the self-error variance of 67%. Hypotheses testing was carried out to see the significance of the variable relationship. The first hypothesis test revealed that (study-related positive emotions → PsyCap) had significant and positive relation (β = 0.532, *t* = 8.629, *p* < 0.05), hence H1 was supported. For the second hypothesis-2, (PsyCap → study engagement), also a significant and positive relationship was observed (β = 0.315, *t* = 4.901 *p* < 0.05), hence H2 was accepted. The direct effect of (study-related positive emotions → on study engagement) was also assessed *via* H3, and it was found that study-related positive emotions had a positive and significant effect on study engagement (β = 0.336, *t* = 5.228 *p* < 0.05), hence H3 was accepted. We also tested the mediate effect of PsyCap between positive emotions and study engagement. Results of bootstrapping revealed that the relationship between (study-related positive emotions → PsyCap → study engagement) was not fully mediated by PsyCap (β = 0.167, *t* = 3.908 *p* < 0.05), as the direct effect of study-related positive emotions on study engagement was statistically significant.

### Moderation Effect Assessment

**FIGURE 2:**
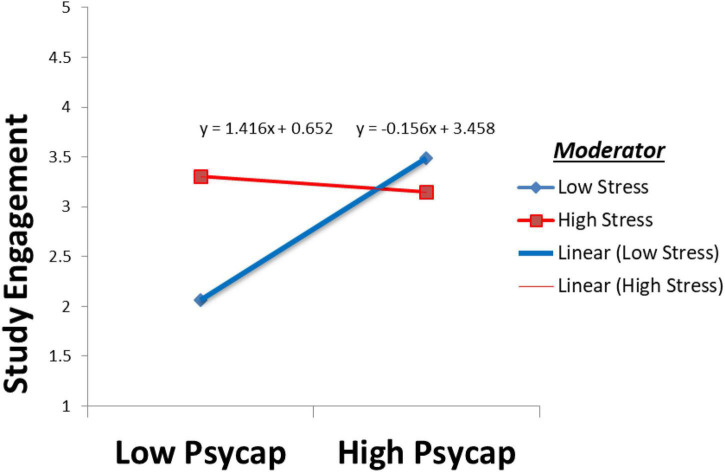
Moderation/interaction effect of stress between psychological capital (PsyCap) and study engagement.

Finally, the outcome of the fourth hypothesis to assess the moderating effect of stress between PsyCap and study engagement revealed significant and negative effects (damping the positive relationship), as expected (β = −0.393, *t* = 5.231 *p* < 0.05), hence H4 was supported. Results of moderation analysis are also shown in Figure 2 for better understanding. All of the aforesaid results are generated through bootstrapping technique at a 95% confidence interval with the 5000 bootstrapping procedure to make the research results robust and reliable, hence we choose to utilize the larger bootstrapping number.

## Discussion

In this study, we evaluated the direct effects of study-related positive emotions on PsyCap and PsyCap’s subsequent effect on study engagement, alongside the assessment of the moderating effect of stress on the relationship between PsyCap and study engagement. According to our findings, study-related positive emotions had a positive effect on PsyCap, and PsyCap was also positively connected to study engagement, hence meeting our theoretical assumptions. Furthermore, results show that a higher degree of stress dampens and decreases the link between students’ PsyCap and their study engagement behavior. It was also found that PsyCap did not completely moderate the relationship between positive emotions and study engagement, albeit with a smaller effect size. According to the literature, mediation analyses are mainly dependent upon their sample size and the difference between complete and partial/no median is very subtle or non-substantive (Hayes, 2013), therefore it is not justified to conclude in a single study to justify mediation or no mediation results. Further research on a different sample with a different contextual variable may yield different outcomes.

Also, our findings are very much pertinent to the past literature, theory, and implications. Theoretically, we highlighted the importance of positive emotions to enhance PsyCap for students, which will further lead them toward their academic engagement behavior. We also highlighted the negative role of stress for the post-graduate students, to see how it can be detrimental to their PsyCap, and decrease their psychological ability to be more engaged in their studies. We have discussed our theoretical contribution, implications, and limitations in later parts.

To explicate the relationship between study-related positive emotions and PsyCap, we used theoretical foundations of broadening and building theory, known as (B&B) (Fredrickson, 2004). Our findings were in harmony with the theoretical assumption that individuals observing positive emotion more often in connection with their academic activities are expected to accumulate positive cognitive resources such as PsyCap, which is consistent with prior literature (Diener et al., 2010; Oriol-Granado et al., 2017; Carmona-Halty et al., 2021). Based on the findings, it can be summarized that the students’ PsyCap can be seen as a vital element to demonstrate enhanced academic engagement. Also, the importance of positive feelings or having positive emotions to generate substantial PsyCap for students can be seen as an important factor. It is not possible to highlight all such factors that promote positive emotions for students, but some of them might include social milieu, personal resolve, Personal self-efficacy, Effective functioning (Phan et al., 2019), feedback Strategies, teacher-student relationship, and collaborative learning (Lai et al., 2020), whereas future research on such elements to foster positive emotions for students in different contexts can generate more insights.

Secondly, to explain the relationship between PsyCap and study engagement, we used the theoretical rationale of job demand resource theory (Bakker and Demerouti, 2014), stating that resources, be it physical or psychological, decrease the demanding situational effects, helping individuals to engender their growth and learning (Bakker and Demerouti, 2007). Based on our findings, the cognitive resources of students, PsyCap has shown a positive effect on academic engagement behavior, another form, and a source of growth and learning for students. Our findings also supported this theoretical assumption and are also consistent with prior literature (Luthans et al., 2012; Carmona-Halty et al., 2021; Kang et al., 2021; Saman and Wirawan, 2021). Keeping this perspective in view, it can be suggested that the accumulation of positive psychological resources in the form of PsyCap can help individuals to enhance their performance not just in academics (Luthans et al., 2014) but in general occupational settings as well (Luthans et al., 2007b,^2012^). Since the construct of PsyCap is based on its four principal components, hope, efficacy, resilience, and optimism (Luthans et al., 2007b), much work is needed to explain their individual and distinct relationship to enhancing student engagement in the educational context.

Thirdly, we used COR to advance the moderation effect of stress on the relationship between PsyCap and students’ engagement behavior. As stated through COR, individuals tend to accumulate resources, be it physical or non-physical to attain goals and objectives, whereas stress occurs if any of the resources loses, or even if there is a fear of losing resources (Hobfoll, 1989). Since stress is classified as one of the psychological hazards (Tagoe and Amponsah-Tawiah, 2019), it tends to deter the resources of the individual. Because of that, individuals may feel burnout, possibly exhausting their cognitive resources (PsyCap), or even pushing them for withdrawing from their role, as well as individuals may not be interested to show engagement behavior. Based on our findings, stress was found to exhibit a negative or deteriorating effect on the relationship between students’ PsyCap and their study engagement behavior. Our findings imply that the more post-graduate student is exposed to or have experienced the stress, they are more likely to lose their cognitive resource, which could have possibly enhanced their academic engagement. There is no doubt that the prevalence of stress for a student concerning their academic activities has long been studied and has shown its negative effect in the past on different cadre of students (Beiter et al., 2015; Vasugi and Hassan, 2019; Pascoe et al., 2020). Nonetheless, our findings are in harmony with prior literature. Since the stress for students results in their decreased well-being and anxiety (Pascoe et al., 2020), therefore specific elements that cause stress for post-graduate students are needed to be identified. The demanding nature of post-graduate studies could be one of the possible reasons for stress for students since students are expected to meet strict timelines with predefined milestones, work-family balance, routine responsibilities, and other financial and non-financial factors (Vasugi and Hassan, 2019). In the future, more refined research inquiries are required to look more specifically into those factors that cause stress for post-graduate students.

## Conclusion

To summarize the aforesaid arguments, we tested all three theoretical assumptions, e.g., positive emotions strengthen one’s broadening and building aspect, and when individuals experience positive emotions, this helps them to build a repository of psychological resources, i.e., PsyCap, which is expected to lead to a stronger will to show academic engagement for post-graduate students. Nonetheless, the negative effect of stress and strain deters the positive effect of one’s PsyCap on his/her study engagement behavior. Our study contributes to the existing body of knowledge by explicating the direct association of positive emotions to foster academic Psycap and exhibits the mediating role of PsyCap to generate study engagement for post-graduate students, whereas the evidence to assess this relationship in a higher academic context is almost non-existent (to authors’ knowledge), therefore this study adds novel dimensions to the understanding. We also testified three different theoretical assumptions and our results met all those assumptions, thus providing more efficacy to the predictability of those theories cited in this research.

### Implications

The primary implication of this research is the impact of positive emotions on post-graduate students. Because students are connected or associated with their field-related supervisors at the post-graduate level, it would be much more beneficial for supervisors to increase their focus on a deep understanding of their student’s needs and expectations (Conrad and Phillips, 1995; Hortsmanshof and Conrad, 2003), as it will help them to provide them with more meaningful inputs/feedback that can ease their post-graduate journey, rather than focusing on providing academic output in the form of papers, achievements, and so on. This deep understanding and consideration by an academic supervisor may result in improved psychological resources for students, alleviating them from the sense of academic and intellectual isolation, giving them more confidence and possibly increasing academic-related engagement. Actions from academic supervisors like giving autonomy and control to their students, setting manageable goals, appropriate follow-up style, and building trust and respect may foster the psychological needs of their students, which in turn benefit both. Also, from the post-graduate student’s side measures such as managing the technological aspect of their studies (effective use of the available knowledge/information, the balance between personal life and professional studies, and the harmony with the given environment may help them to generate positive emotions and sanity to furnish their journey.

Regarding the effect of PsyCap and academic engagement, academic supervisors of post-graduate students can work upon this, by giving them hope (showing positive paths and favorable futuristic outcomes), efficacy (providing them confidence through learning and development opportunities), resilience (supporting them, while they lack or fall behind), and optimism (emphasizing more on positive appraisals of the events throughout the academic journey). Since strong psychological conditions are necessary to cope with overwhelming tasks, therefore teachers’ understanding of the psychological aspects while dealing with post-graduate students would be an added value for students. Intervention at the university management level to strengthen the positive psychology of students might help achieve this task for teachers/faculty.

Finally, keeping the negative role of stress in mind for post-graduate students, teachers, and university administration can play an important role in reducing this effect. Because post-graduate supervisors work closely and personally (often) with their students, it would be extremely beneficial for students if their supervisors could have a thorough understanding of their student’s needs and demands at specific times and periods. More informal discussion and friendliness would help strengthen teachers’ understanding of students’ current mental states, allowing them to better strategize study goals and objectives. Further, for post-graduate students stress can be mitigated by opting to work in supportive peer groups (Wisker et al., 2007), which will help build momentum and may also provide social support. At the organizational level, interventions such as formally assessing psychological stress through surveys and training can also assist students in determining their current level of stress and appropriate coping strategies. Academic organizations should design their study milestone according to the best fit between available resources and desired outcomes.

### Study Limitations

One of the first limitations of this study is the usage of the cross-sectional approach, as the longitudinal approach adding more samples to the study would yield different interesting insights. Secondly, our respondents may have faced the social-desirability bias (Braun et al., 2001) while reporting their emotions, PsyCap, and academic engagement a common phenomenon reported in past studies also (Naji et al., 2022a,b). Although we took measures like keeping the survey anonymous, and confidential, and not asking for the names or identities of respondents in any form, but still there is a tendency for individuals to rate themselves for generally acceptable behavior on a higher side and rate themselves on a lower side for undesirable/socially in appreciated actions/norms. Another limitation of our study is our population being studied, as a considerable size of respondents, i.e., post-graduate students were from other nations who have traveled to this country for their post-graduate studies, therefore our sample does not reflect entirely the local population, but it includes other nationality as well. This may have affected to some extent our findings as foreign students are living in different circumstances with different educational, family, financial, and other backgrounds. Therefore, a study based on entire local post-graduate students may yield different outcomes, based on the difference in difficulties and situations faced by foreign post-graduate students.

## Data Availability Statement

Raw data of this research are only available upon reasonable request through corresponding author.

## Ethics Statement

Ethical review and approval was not required for the study in accordance with the local legislation and institutional requirements. The participants provided their written informed consent to participate in this study.

## Author Contributions

MS, MA, and GN worked on idea inception, writing up the draft and finalizing the findings, and overall manuscript. AI and YY substantial contribution to the conception, revising and reviewing manuscript critically. All authors contributed to the article and approved the submitted version.

## Conflict of Interest

The authors declare that the research was conducted in the absence of any commercial or financial relationships that could be construed as a potential conflict of interest.

## Publisher’s Note

All claims expressed in this article are solely those of the authors and do not necessarily represent those of their affiliated organizations, or those of the publisher, the editors and the reviewers. Any product that may be evaluated in this article, or claim that may be made by its manufacturer, is not guaranteed or endorsed by the publisher.
